# Subclavian Vein Thrombosis in a Patient With Venous Thoracic Outlet Syndrome and Previous Preventive First Rib Resection

**DOI:** 10.7759/cureus.84315

**Published:** 2025-05-18

**Authors:** Maria R Kuzmanova, Lucas L. Rau, Ulrike Hügel, Nils Kucher, Stefano Barco

**Affiliations:** 1 Angiology, University Hospital Zurich, Zurich, CHE

**Keywords:** angiology, cardiovascular intervention, thoracic outlet, tos, vascular occlusion

## Abstract

Deep vein thrombosis (DVT) associated with venous thoracic outlet syndrome (vTOS) after rib resection is rare. In younger, physically active people, repetitive upper extremity activity can lead to effort thrombosis (Paget-Schroetter syndrome), as the subclavian vein is chronically injured and becomes fibrotic. Without restored patency, venous collaterals form as a compensatory mechanism to overcome chronic occlusion.

We report a successful mini-invasive endovascular thrombectomy for right subclavian vein thrombosis in a 33-year-old female patient with bilateral first rib resection performed at another hospital 10 years prior, which was performed as a secondary thrombosis prevention on the left side and as primary thrombosis prevention on the right, non-acutely thrombosed side.

We discuss key therapeutic aspects, including anticoagulation duration, need for reintervention, and indication for stent placement after rib resection.

## Introduction

The thoracic outlet syndromes (TOSs) are characterized by the compression of neurovascular structures located in the superior thoracic aperture [1,2]. Venous TOS (vTOS) is diagnosed based on an anatomical constriction of the vein between the first rib and the clavicle leading to typical symptoms, including but not limited to venous claudication, chronic swelling, and vein thrombosis.

It is estimated to occur in at least eight cases per 1,000,000 population-years. The estimated overall incidence of TOS varies widely, suggesting that it may be underrepresented in current medical literature [3].

In some young, sporty patients, effort thrombosis, also known as Paget-Schroetter syndrome, may affect a chronically injured subclavian vein because of repetitive and strenuous activity of the upper extremities [2]. Anatomical predisposition and thrombophilia may play an additional role [4]. 

If the patency of the vein cannot be restored, post-thrombotic syndrome is a well-described complication if the collaterals do not suffice to restore adequate outflow. The patient's history and physical examination maneuvers are often typical and easy to recognize. Imaging tests would confirm the presence of a vein narrowing. In patients with acute deep vein thrombosis (DVT), there is ongoing discussion on the role of early endovascular reperfusion with or without first rib resection with scalenotomy to relief the chronic compression [5]. Preventive surgery for subclavian thrombosis has been rarely described: its efficacy and safety remain unproven [6]. 

## Case presentation

In 2007, an 18-year-old female patient experienced a first episode left subclavian vein thrombosis secondary to vTOS. The patient was in good health, without cardiovascular risk factors, comorbidities, or genetic/acquired thrombophilia. After having experienced a recurrent episode under poorly controlled vitamin K antagonist anticoagulation a few months later, a bilateral first rib resection was performed at another center as a secondary prevention on the left side and a primary prevention on the non-thrombosed right side. The surgical approach used in the preventive first rib resection and whether a scalenectomy was done is unclear. Two years later, anticoagulation was discontinued and the patient experienced no further recurrent thrombotic events for more than a decade. 

Fifteen years later, the patient was diagnosed with acute, symptomatic right-sided upper-extremity DVT associated with a segmental pulmonary embolism, which occurred after climbing an 8,000-meter mountain. Main symptoms were acute swelling and pain. She was initially started on dalteparin at an unclear dosage and later switched to therapeutic-dosed rivaroxaban. 

Two weeks later, due to persistent swelling and pain in the right upper extremity, the patient was referred to our center. Upon physical examination, swelling in the right upper extremity without skin discoloration or lesions and superficial collateral veins were present. 

**Figure 1:**
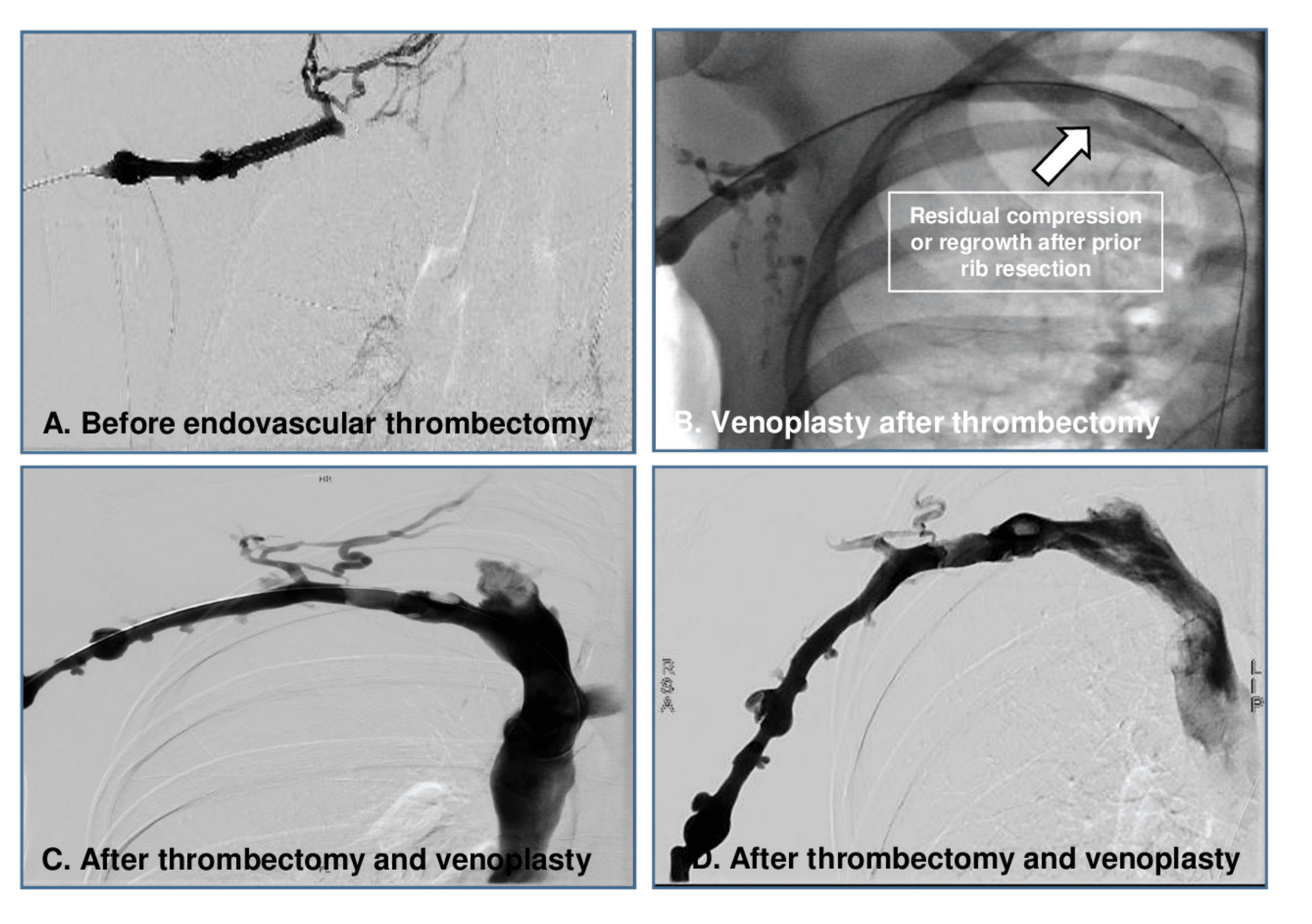
MRI angiography and duplex sonography before and after thrombectomy A) Residual compression or regrowth after prior rib resection; B) Before endovascular thrombectomy; C) After endovascular thrombectomy; D) After endovascular thrombectomy

**Figure 2:**
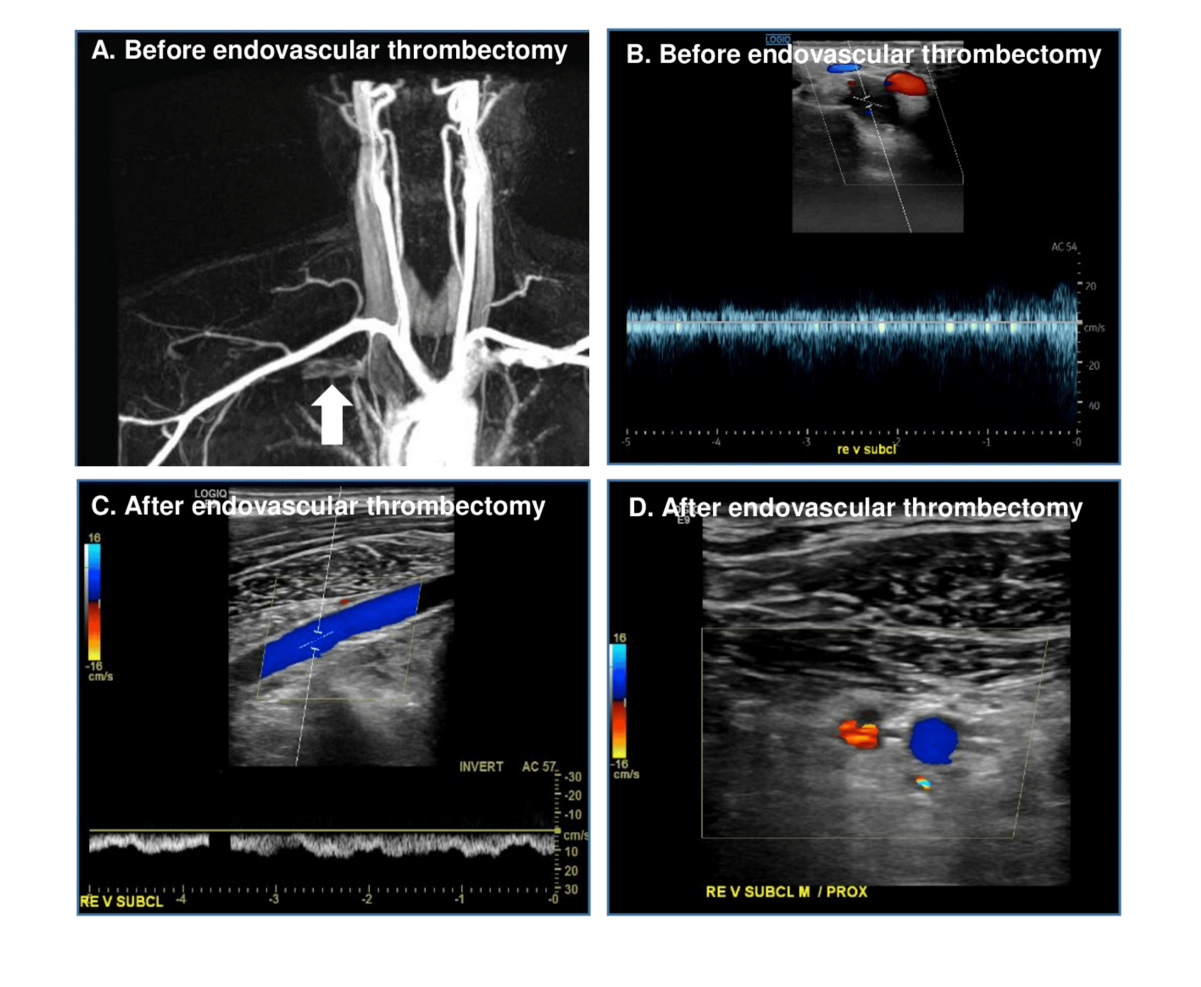
DAS before and after venoplasty A) Before endovascular thrombectomy; B) Venoplasty after thrombectomy; C) After thrombectomy and venoplasty; D) After thrombectomy and venoplasty DAS: Digital subtraction angiography

Pre-interventional magnetic resonance venography and color-coded duplex sonography showed proximal subclavian vein short-segment (2.5 cm) thrombosis, leading distally to a band-shaped flow pattern (Figure 1, Panels A-B). Of note, pre-interventional magnetic resonance venography showed no apparent regrowth/residual of the surgically removed first rib on both sides. A pharmacomechanical thrombectomy with AngioJet PowerPulse (Alteplase 10 mg) followed by Penumbra 7-French Indigo thrombectomy due to insufficient flow restoration and venoplasty after complete thrombus removal were performed via an endovascular approach through basilic vein without complications. Figure 2 (Panels A-B) shows the initial contrast enhancement before endovascular thrombectomy and, thereafter, a residual flaw contrast enhancement in the site of chronic vein compression (Figure 2, Panels C-D) is seen, indicating a fibrous transformation and thickening of the vein wall. During follow-up, the subclavian vein remains almost completely recanalized with no visible collaterals (Figure 1, Panels C-D). Upon discharge, the patient was prescribed with therapeutic-dosed rivaroxaban and compression stockings. Further thrombophilia work-up was normal. Six months later, the vein was still patient, though with post-thrombotic alterations on the wall without evidence of a residual thrombus. The patient remained asymptomatic during the whole follow-up. Over long-term, we suggested secondary thrombosis prevention with reduced-dosed oral anticoagulants vs. reintervention (re-do after rib resection).

## Discussion

We report a rare case of contralateral subclavian vein thrombosis occurring ten years after a first rib resection performed for primary prophylaxis of vTOS. To our knowledge, this is the first documented instance of such a delayed complication following preventive surgical decompression. The acute thrombosis was successfully treated with a minimally invasive, endovascular approach.

In this physically active mountaineer, exertion-related physiological changes may have played a contributing role. Intense exercise is known to induce a transient hypercoagulable state, and although fibrinolysis is simultaneously activated, the net effect depends on individual and activity-related factors [7].

The underlying mechanism of re-thrombosis remains uncertain. However, angiographic findings during venoplasty suggested residual fibrosis of the subclavian vein, likely contributing to chronic venous outflow impairment and endothelial dysfunction. This case highlights the potential role of post-surgical fibrotic remodeling in long-term thrombotic risk and underscores the need for further investigation into such delayed complications.

Recurrent symptoms of vTOS occur in 15% to 20% of patients who undergo either first rib resection or scalenectomy for TOS, while 70% experience symptom improvement [8]. The remaining 30% seem to need further investigation. A regrowth of the first rib is seen in rare cases. One study showed that 10 of 726 surgical interventions performed in 551 patients were for resection of re-grown first ribs: regrown ribs accounted for 10.6% of surgeries for recurrent TOS symptoms, 1.4% of all patients, and 1.1% of all procedures [9]. Furthermore, there is a possibility of multiple compression sites in patients with vTOS. Incomplete surgical release of all compression points leaves patients prone to re-thrombosis and/or persistent post-thrombotic syndrome. Timely recognition of all abnormalities on venography may allow for adjustment of surgical treatment accordingly [10]. 

In our case, we showed that pre-interventional magnetic resonance venography showed no residual compression of the subclavian vein. After thrombectomy and venoplasty, we documented chronic changes (i.e. fibrosis) of the vein wall. This indicates that a vessel remains prone to rethrombosis, particularly in the case of additional contributing factors, like strenuous exercise and prolonged compression due to wearing of a backpack. Optimal follow-up management in these patients, including dose/length of anticoagulation, (re)-intervention, stent placement after rib resection, remains matter of discussion. The therapeutic approach should be discussed interdisciplinary from the very beginning. 

## Conclusions

This case illustrates a rare but important delayed complication of vTOS-contralateral subclavian vein thrombosis occurring a decade after prophylactic first rib resection. The successful management with a minimally invasive endovascular approach highlights the effectiveness of contemporary treatment strategies for late-presenting thrombotic events. Our findings suggest that chronic vein wall changes, such as fibrosis, may predispose to thrombosis even in the absence of residual anatomical compression, particularly when compounded by physiological stressors like intense physical activity.
